# A Genome-Wide Screen to Identify Transcription Factors Expressed in Pelvic Ganglia of the Lower Urinary Tract

**DOI:** 10.3389/fnins.2012.00130

**Published:** 2012-09-12

**Authors:** Carrie B. Wiese, Sara Ireland, Nicole L. Fleming, Jing Yu, M. Todd Valerius, Kylie Georgas, Han Sheng Chiu, Jane Brennan, Jane Armstrong, Melissa H. Little, Andrew P. McMahon, E. Michelle Southard-Smith

**Affiliations:** ^1^Division of Genetic Medicine, Department of Medicine, Vanderbilt University School of MedicineNashville, TN, USA; ^2^Department of Cell Biology, University of Virginia School of MedicineCharlottesville, VA, USA; ^3^Department of Surgery, The Transplant Institute, Beth Israel Deaconess Medical Center, Harvard Medical SchoolBoston, MA, USA; ^4^Institute for Molecular Bioscience, University of QueenslandBrisbane, QLD, Australia; ^5^GUDMAP Editorial Office, Center for Integrative Physiology, University of EdinburghEdinburgh, UK; ^6^Department of Stem Cell and Regenerative Biology, Harvard Stem Cell Institute, Harvard UniversityCambridge, MA, USA; ^7^Department of Molecular and Cellular Biology, Harvard Stem Cell Institute, Harvard UniversityCambridge, MA, USA

**Keywords:** pelvic ganglia, autonomic nervous system, transcription factor, *in situ* hybridization, lower urinary tract, genitourinary, mouse, Genitourinary Development Molecular Anatomy Project

## Abstract

Relative positions of neurons within mature murine pelvic ganglia based on expression of neurotransmitters have been described. However the spatial organization of developing innervation in the murine urogenital tract (UGT) and the gene networks that regulate specification and maturation of neurons within the pelvic ganglia of the lower urinary tract (LUT) are unknown. We used whole-mount immunohistochemistry and histochemical stains to localize neural elements in 15.5 days post coitus (dpc) fetal mice. To identify potential regulatory factors expressed in pelvic ganglia, we surveyed expression patterns for known or probable transcription factors (TF) annotated in the mouse genome by screening a whole-mount *in situ* hybridization library of fetal UGTs. Of the 155 genes detected in pelvic ganglia, 88 encode TFs based on the presence of predicted DNA-binding domains. Neural crest (NC)-derived progenitors within the LUT were labeled by *Sox10*, a well-known regulator of NC development. Genes identified were categorized based on patterns of restricted expression in pelvic ganglia, pelvic ganglia and urethral epithelium, or pelvic ganglia and urethral mesenchyme. Gene expression patterns and the distribution of Sox10+, Phox2b+, Hu+, and PGP9.5+ cells within developing ganglia suggest previously unrecognized regional segregation of Sox10+ progenitors and differentiating neurons in early development of pelvic ganglia. Reverse transcription-PCR of pelvic ganglia RNA from fetal and post-natal stages demonstrated that multiple TFs maintain post-natal expression, although *Pax3* is extinguished before weaning. Our analysis identifies multiple potential regulatory genes including TFs that may participate in segregation of discrete lineages within pelvic ganglia. The genes identified here are attractive candidate disease genes that may now be further investigated for their roles in malformation syndromes or in LUT dysfunction.

## Introduction

Autonomic innervation of the urogenital tract (UGT) controls many fundamental functions including release of hormones from the adrenal, regulation of renal blood flow, peristalsis of urine along the ureters to the bladder, and coordination of urine release from the bladder, as well as sexual arousal. These critical functions can be disrupted by age, disease, or surgical interventions. Anatomy of these neural structures is essential for urological training and efforts to readily visualize these critical elements have been a focus in recent years (Lee et al., 2010; Taylor et al., 2010).

Anatomical study of urogenital autonomic ganglia and their accompanying peripheral nerve fibers has been achieved primarily in adult human biopsies and fetal tissues (Jen et al., 1995; Dixon et al., 1998; Yucel et al., 2004). These human studies are challenged by consistency in collection, availability of multiple samples at equivalent developmental stages, and comparison of genetically heterogeneous individuals. However, they have succeeded in defining the location of nerve fibers in the developing urethral sphincter (Yucel et al., 2004; Karam et al., 2005; Wallner et al., 2009), and mapping neuromuscular junctions as well as generally localizing neuronal and glial elements (Kluck, 1980; Wadhwa and Bijlani, 1983; Elbadawi, 1991; Yucel et al., 2004).

Pelvic innervation in humans consists of a diffuse pelvic plexus, while that of rodents consists of large paired pelvic ganglia that flank the urethra. The ready ability to visualize pelvic ganglia in mice has led to detailed neurochemical and anatomical analyses aimed at identifying the locations and distributions of discrete neuronal subtypes within pelvic neural elements in rodents (Yan and Keast, 2008). Such efforts have defined the topography of nerve fibers and defined differential reactivity to neuronal antigens including acetylcholinesterase (AChE), dopamine β-hydroxylase (DβH), tyrosine hydroxylase (TH), ubiquitin carboxy-terminal hydrolase L1 (PGP9.5), neuronal nitric oxide synthase (nNOS), vasoactive intestinal peptide (VIP), and neuropeptide Y (NPY) within the bladder wall and adjacent pelvic ganglia (Wanigasekara et al., 2003; Keast, 2006; Yan and Keast, 2008; Girard et al., 2010). As a result neurotransmitter expression within subsets of pelvic ganglia neurons is known and some of the physiological properties of pelvic ganglia based on immunohistochemical coding of multiple neurotransmitters have been defined (Keast, 2006; Jobling and Lim, 2008; Tompkins et al., 2010).

Unfortunately, the factors that control the development of pelvic innervation are much less well understood. Neurotrophins and glial cell-derived neurotrophic factors do affect cell migration and neurite outgrowth from pelvic ganglia neurons like they do elsewhere in the peripheral nervous system, although initial studies suggest their effects are age and neuron-type dependent (Stewart et al., 2008). However, nothing is known about regulatory networks that control specification of pelvic neuron subtypes or regulate their post-natal maturation. Advances in regenerative strategies to repair damaged pelvic neural inputs are much more likely to be achieved with greater knowledge of processes integral to normal development of pelvic innervation.

High throughput screens to visualize expression of potential transcriptional regulators or receptors and their ligands are one means to identify key regulatory molecules during organogenesis (Gray et al., 2004; Choi et al., 2006; Kong et al., 2006; Mugford et al., 2009). To discover gene networks most likely to control pelvic innervation during development, we surveyed a genome scale whole-mount *in situ* hybridization (WISH) library of fetal mouse UGT hybridized to probes for 1379 genes including 921 transcription factors (TFs) annotated in the mouse genome (Gray et al., 2004). We complemented the survey by identifying the locations of developing neurons and glia in whole-mount fetal UGT. Our study has identified a subset of genes that are expressed in the developing pelvic ganglia, some of which maintained their expression into post-natal maturation. This analysis identifies urological candidate disease genes and lays a foundation for future study of TF networks and gene expression levels in congenital lower urinary tract (LUT) defects, urological disease, and aging.

## Materials and Methods

### Animals

All animal protocols were approved by the Institutional Animal Care and Use Committee at Vanderbilt University. Timed matings were set to obtain staged mouse embryos, designating the morning of plug formation as 0.5 day post coitus (dpc). Post-natal stages were designated with the date of birth as P0 and P2 as the second day after birth. Swiss Webster outbred mice were used to obtain embryos for whole-mount and sectional *in situ*. *Sox10*-H2BVenus mice were maintained as congenic lines on the C3HeB/FeJ inbred strain and bred as previously described to obtain staged litters (Corpening et al., 2011). *Phox2b*-H2BCFP mice were maintained by crosses to C57BL/6J × C3HeBFeJ F_1_ animals (Corpening et al., 2008).

### Whole-mount immunohistochemistry of fetal LUT

Whole-mount immunohistochemistry (IHC) methods were modified from the previously published protocols (Mark et al., 1993; Bamforth et al., 2001; van de Putte et al., 2007). Briefly, fetal LUTs were dissected from 15.5dpc wild-type mice and fixed at 4°C in 4% paraformaldehyde overnight. Endogenous peroxidase activity was inactivated with 3% H_2_O_2_, 80% methanol, and 20% dimethylsulfoxide (DMSO) solution for 3 h. Washes were performed with Tris-buffered saline (TBS) containing 1% Tween-20 and blocked in TBS containing 1% Tween-20 and 5% skim milk. Samples were incubated with either rabbit anti-PGP9.5 (1:4000, Biogenesis), mouse monoclonal anti-2H3/neurofilament (NF; 1:100, Developmental Studies Hybridoma Bank), sheep anti-TH (1:100, Chemicon), or rabbit anti-vesicular acetylcholine transporter (VAChT; 1:1000, Synaptic Systems) antibody diluted in block containing 5% DMSO and 0.1% sodium azide for 3 days at room temperature. Primary antibodies were detected with HRP-conjugated donkey anti-rabbit IgG (1:1000), HRP-conjugated donkey anti-mouse IgG (1:100), and HRP-conjugated donkey anti-sheep IgG (1:200), respectively (Jackson Immuno Research). Visualization was achieved with 4-chloro-1-naphthol solution (Sigma-Aldrich) according to the manufacturer’s instructions.

### Sectional embryonic immunohistochemistry

Whole-mount 15.5dpc *Sox10*-H2BVenus+ and *Phox2b*-H2BCFP+ double transgenic embryos were fixed at 4°C in neutral buffered formalin for 5 h and washed with 1× PBS before incubating in 30% sucrose at 4°C overnight. The samples were cryo-embedded by rapid freezing on dry ice after submerging into OCT tissue freezing media (Tissue Tek). A Lecia CM1900 cryostat was utilized to generate 18 μm thick sections onto treated slides, which were heated on a slide warmer for 30 min. Melted freezing media was removed by washes in 1× PBS + 0.5% Triton X-100 and the sections were blocked for 1 h at room temperature in 1× PBS containing 0.1% Triton X-100, 5% heat-inactivated normal donkey serum (Jackson Immuno Research), and 1% bovine serum albumin. Sections were incubated overnight at 4°C with Hu (Kiers et al., 1991; Fairman et al., 1995, 1:800) and rabbit anti-PGP9.5 (1:4000, Biogenesis) antibodies diluted in block. Primary antibodies were detected with texas red-conjugated donkey anti-human (1:100, Jackson Immuno Research) and Cy5-conjugated donkey anti-rabbit (1:400, Jackson Immuno Research). Sections were incubated with secondary antibodies for 1 h at room temperature, followed by 1× PBS washes then mounted in Aqua-Poly/Mount (Polysciences, Inc.).

### Acetylcholinesterase staining

Fetal LUTs were dissected from 15.5dpc wild-type mice and whole-mount AChE enzyme histochemistry was performed essentially as previously described (Enomoto et al., 1998).

### *In situ* hybridization

Whole-mount and sectional *in situ* hybridization were performed as previously described (Little et al., 2007; Mugford et al., 2009) and as listed in detail on the GUDMAP gene expression database (http://www.gudmap.org; see http://www.gudmap.org/research/protocols/McMahon.html and http://www.gudmap.org/research/protocols/Little.html for experimental details). Full details of the screen for mammalian TF expression are described elsewhere (Yu et al., 2012).

### Imaging

Whole UGT samples were further dissected after colorimetric development to facilitate imaging of the LUT. Samples were imaged in whole-mount using a DAGE DC330 Camera mounted on a Leica MZ12.5 stereomicroscope in either dark field or bright field views. Images were taken at either 25 or 50× magnification. Images were limited to 300 dpi, cropped in Adobe Photoshop and adjusted for brightness and contrast to provide the best two-dimensional representation of each gene expression pattern. *Sox10*-H2BVenus whole-mount bladder and urethra images were captured using Zeiss Stereo Lumar.V12 fluorescence stereomicroscope equipped with a Q-Imaging 4000R digital camera and software. The lateral images of 14.5dpc, 15.5dpc, P2, P10, P21 LUT were taken at 25–50× magnification. Confocal microscopy of sectional IHC was performed on a Zeiss Scanning Microscope LSM510 using CFP, YFP Rhodamine, and Cy5 long pass filters as previously described (Corpening et al., 2008, 2011) to visualize transgenic expression of CFP and YFP in combination with neuronal antigens detected by antibodies.

### Reverse transcription-PCR

Using a Zeiss Stereo Lumar.V12 fluorescence stereomicroscope, YFP positive pelvic ganglia were dissected from *Sox10*-H2BVenus transgenic mice then stored in RNAlater at 4°C. To ensure sufficient RNA yield from tissue isolates at each stage pelvic ganglia were pooled from 6, 5, 2, 4, and 3 individual animals at 14.5dpc, 15.5dpc, P2, P10, and P21, respectively. Total RNA was purified from isolated pelvic ganglia by homogenizing tissues in TRIzol (Invitrogen) and extracting RNA as previously described (Iwashita et al., 2003; Corpening et al., 2011). RNA was treated with DNase I and purified via RNeasy kit (Qiagen). Reverse transcription (RT) was performed using a high capacity cDNA RT kit (ABI) with a 50 μL reaction and starting input of 160 ng of RNA. Subsequently cDNA (3 ng input per reaction) was amplified using routine PCR conditions. PCR was done with limiting numbers of thermocycles (<30) during exponential phases of amplification to provide an estimate of relative gene expression with all the reactions for the different stages of a particular gene assay performed and evaluated in parallel. RT-PCR product sizes were evaluated by non-denaturing polyacrylamide gel electrophoresis.

## Results

### Whole-mount visualization of neural elements in the fetal mouse UGT

**Figure 1:**
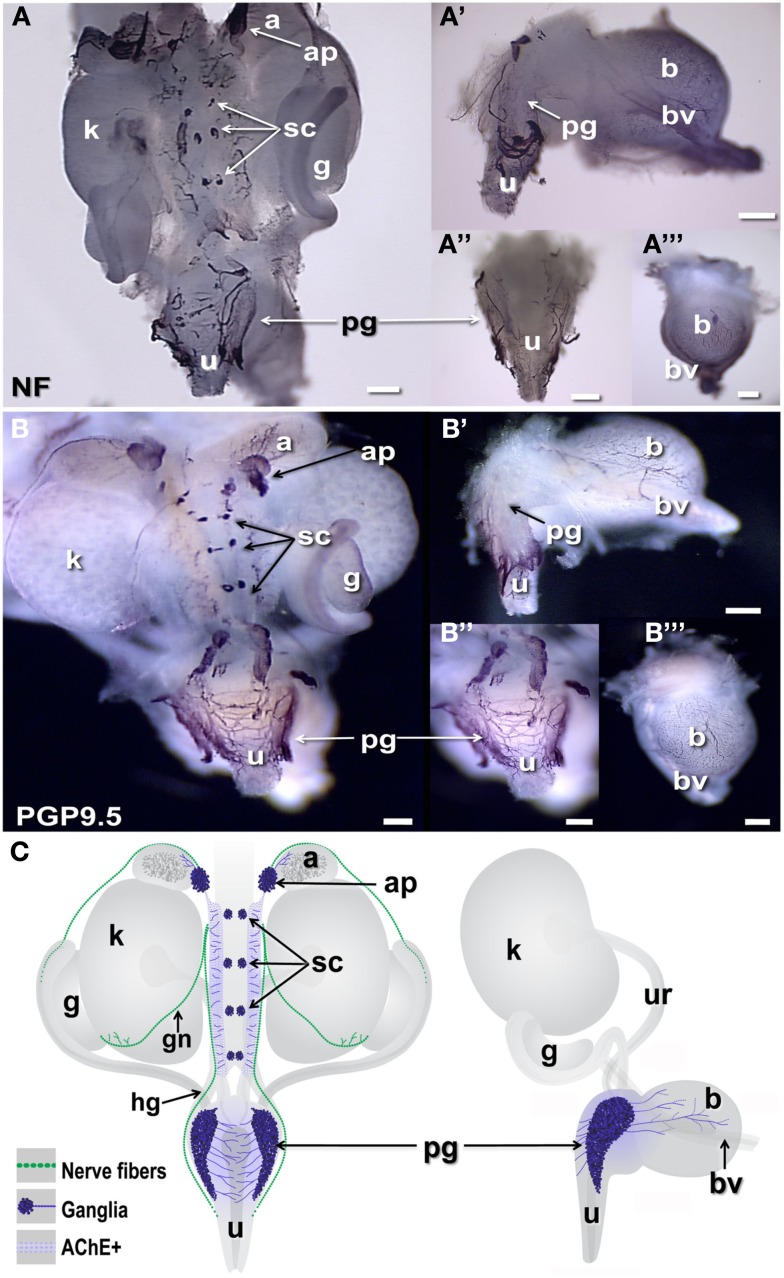
**Distribution of neural elements in fetal mouse LUT**. Dorsal, lateral, and anterior views of whole-mount urogenital tract (UGT) processed to detect neuronal antigens are shown. **(A)** Positions of peripheral ganglia and nerve fibers are labeled by whole-mount IHC in female 15.5dpc fetal LUT stained for neurofilament (NF). **(B)** Whole-mount IHC for PGP9.5 similarly stains both peripheral ganglia and nerve processes at this stage in female LUT. Dorsal views of the intact UGT are shown for each antigen. Lateral views of dissected bladder with attached urethra are shown **(A′,B′)**. Dorsal views of the urethra **(A″,B″)** and anterior views of the bladder (**A‴,B‴**) show relative positions of the pelvic ganglia to these structures. **(C)** Schematic diagrams of fetal mouse UGT at 15.5dpc summarize elements of autonomic innervation detected by IHC in dark blue. Axonal processes of efferent innervation that are accompanied by NC-derived peripheral glia are shown in green. In dorsal views (left), the adrenal plexus extends processes into the NC-derived medulla (dark gray) of the adrenal. Sympathetic chain ganglia at this stage reside as tight clusters medially between the kidneys. Neurites from bilateral pelvic ganglia flanking the urethra extend processes toward the center of the dorsal urethra. When viewed laterally (right) neurites also extend from pelvic ganglia and out toward the bladder dome along blood vessels that flank the bladder. (a, adrenal; ap, adrenal plexus; b, bladder; bv, blood vessel; g, gonad; gn, gonadal nerve; hg, hypogastric nerve; k, kidney; pg, pelvic ganglia; sc, sympathetic chain; u, urethra; ur, ureter; scale bar = 300 μm).

The distribution of neural elements in the fetal murine UGT has not previously been described in whole-mount. Typically images of these structures are derived from small pieces of dissected material or tissue sections subjected to IHC to reveal the positions of particular cell-type specific antigens. However, axonal projections are not always evident in single sections, thus to establish a baseline for identifying genes expressed in pelvic neural elements and gain an overall spatial perspective of condensing autonomic ganglia, we implemented whole-mount immunohistochemical detection using immunoreagents to detect neuronal cell types including Neurofilament (NF) and Protein gene product 9.5 (PGP9.5). NF comprises a major element of the axonal cytoplasm in differentiated neurons so that detection of this antigen by IHC labels cell processes. PGP9.5 protein, which derives from the gene *Uchl1*, is an ubiquitin hydrolase that is found both in the cell soma as well as in axonal processes (Thompson et al., 1983; Young et al., 2003; Corpening et al., 2011). While PGP9.5 is often used as generic marker to identify mature neurons, it also labels cells in early phases of neuronal differentiation in both the central and peripheral nervous systems (Sidebotham et al., 2001; Sakurai et al., 2006; D’Autreaux et al., 2007). The IHC distribution of NF and PGP9.5 revealed discrete clusters of developing neurons in the forming sympathetic chain at the dorsal mid-line of the UGT (Figures 1A,B). Fibrous staining of axonal processes extending from these condensations laterally was evident medial to the kidneys and at more rostral levels near the adrenals. At the level of the urethra, staining of axonal processes extending from the pelvic ganglia that wrapped dorsally behind the urethra was also strongly labeled by both NF and PGP9.5. Moreover a dense network of PGP9.5 reactive processes was already evident in the bladder wall at 15.5dpc extending toward the bladder dome in both lateral and anterior views (Figures 1A′,A‴,B′,B‴). A summary diagram depicting the distribution of neural elements detected in the UGT relative to the developing fetal organs at 15.5dpc is provided in Figure 1C.

**Figure 2:**
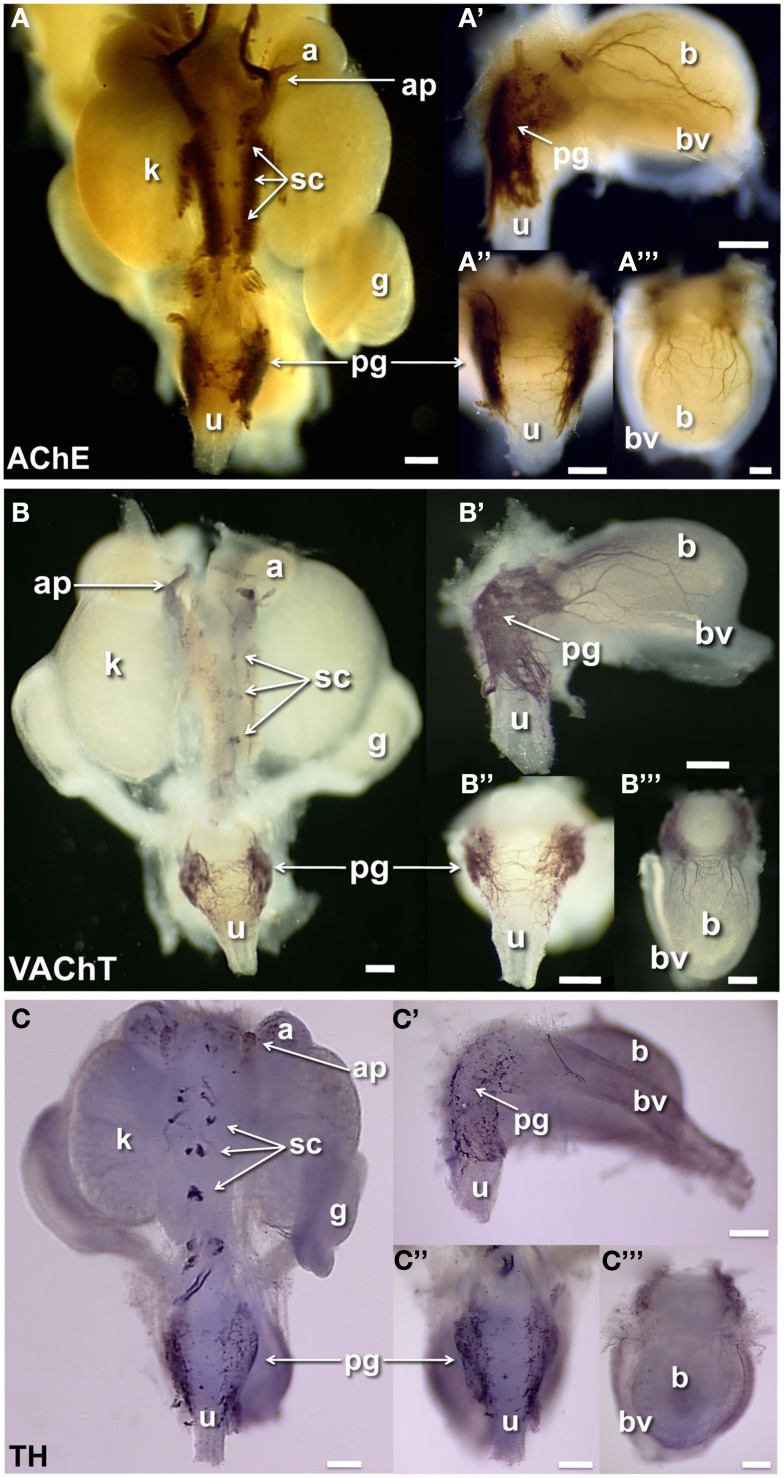
**Distribution of cholinergic and noradrenergic elements in fetal mouse LUT**. Whole-mount images of male 15.5dpc fetal UGT samples stained by AChE histochemistry localizes primarily cholinergic innervation and some other neuronal subtypes **(A)**. IHC staining of female 15.5dpc fetal LUT detects specifically cholinergic VAChT+ neurons **(B)**. IHC staining of female LUT detects sympathetic noradrenergic innervation labeled by tyrosine hydroxylase immunoreactivity **(C)**. **(A–C)** Consist of whole-mount images of 15.5dpc urogenital tissues including dorsal views **(A–C)**, lateral views of bladder **(A′–C′)**, dorsal views of urethra **(A″–C″)**, and anterior view of bladder **(A‴–C‴)**. (a, adrenal; ap, adrenal plexus; b, bladder; bv, blood vessel; g, gonad; k, kidney; pg, pelvic ganglia; sc, sympathetic chain; u, urethra; scale bar = 300 μm).

Prior studies have reported extensive cholinergic innervation in sectioned UGT tissues of adult mice (Yan and Keast, 2008). To begin identifying the positions of developing cholinergic neurons, we initially applied whole-mount acetylcholinesterase (AChE) histochemistry, a robust detection method that has been used extensively to visualize cholinergic neurons within enteric ganglia of the intestinal wall (Enomoto et al., 1998). AChE strongly labels neuronal cell bodies as well as their processes but is not restricted to cholinergic neurons (Soreq and Seidman, 2001). AChE histochemistry extensively labeled axonal processes and ganglia in the fetal UGT at 15.5dpc (Figure 2A). Processes connecting the adrenal ganglia, sympathetic ganglia, and kidney pelvis were strongly labeled as well as neuronal bodies within the pelvic ganglia flanking the urethra. The bladder wall at 15.5dpc contained large numbers of AChE+ neural fibers that were primarily evident in lateral areas adjacent to the flanking blood vessels. This distribution contrasts to the much broader distribution in the bladder dome detected by PGP9.5 IHC and suggests either that PGP9.5 reactivity detects a population of early differentiating neurons expressing a different neurotransmitter in the medial anterior bladder wall or that accumulation of AChE activity has not reached sufficient levels for detection by this staining method.

To visualize specific neuronal populations in the developing UGT, we examined the distribution of cholinergic neurons labeled by VAChT and noradrenergic neurons labeled by TH (Wanigasekara et al., 2003). VAChT co-localizes with choline acetyltransferase in cholinergic neurons and has been used extensively as a surrogate marker for cholinergic neurons (Arvidsson et al., 1997; Ichikawa et al., 1997). VAChT was detected in the forming ganglia of the sympathetic chain and within dorsal celiac ganglia flanking the mid-line between the developing kidneys (Figure 2B). At 15.5dpc pelvic ganglia were strongly VAChT+ and numerous VAChT+ fibers were present in the dorsal aspect of the urethra. Numerous VAChT+ fibers were also evident in the bladder in both lateral and anterior views (Figures 2B,B′,B‴). While VAChT was observed only in the adrenal plexus, TH immunoreactivity labeled both the adrenal medulla and as well as noradrenergic neurons of the adrenal plexus (Figure 2C). Noradrenergic neurons labeled by TH immunoreactivity were also detected in the forming ganglia of the sympathetic chain and in isolated cells throughout the pelvic ganglia as well as in a few scattered cell bodies at the dorsal aspect of the urethra. While VAChT had extensively labeled fibers in the developing bladder, TH reactivity in the bladder wall, was barely discernable when viewed in whole-mount. TH+ cells were faintly visible in lateral and anterior views (Figures 2C′,C‴) and were observed most frequently in the lateral bladder wall near the flanking blood vessels unlike the labeling for NF and PGP9.5 that was prominent in lateral areas of the bladder as well as the forming bladder dome.

**Figure 3:**
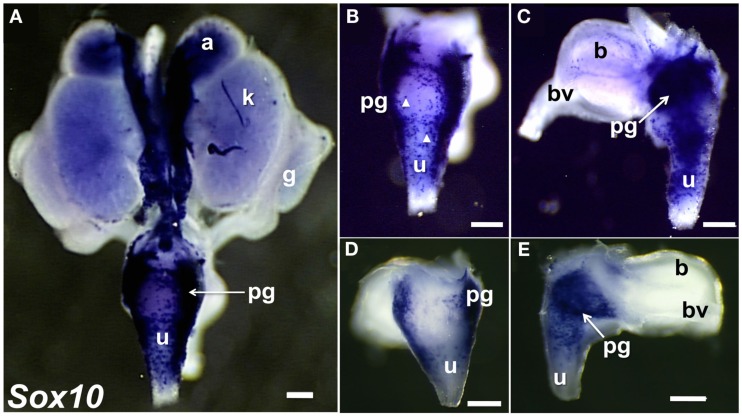
**Distribution of Sox10+ NC-derived progenitors in fetal mouse LUT**. Whole-mount images of 15.5dpc UGT hybridized with anti-sense probes to detect *Sox10* expression are shown. NC-derived cells are strongly labeled in dorsal view of entire UGT **(A)**, dorsal view of urethra **(B)**, and lateral view of bladder **(C)**. Arrowheads highlight stained single cells visible in the dorsal view of the urethra and lateral view of the bladder. (GUDMAP Probe MTF511, MGI:3506241, scale bar = 300μm). Images of 15.5dpc UGT samples developed for a shorter period of time to discriminate regions of high versus low Sox10 expression are shown **(D,E)**. Sox10+ progenitors are present in dorsal urethra [**(D)**, 50×] and in pelvic ganglia viewed laterally **(E)**. Note the high intensity of Sox10 expression within the interior region of the pelvic ganglia (arrow). GUDMAP probe MTF511, MGI:3506241. Scale bar = 300 μm.

To visualize neural crest (NC)-derived progenitors, we applied WISH to localize *Sox10* expression in 15.5dpc fetal LUT (Figure 3). *Sox10* is a TF that is essential for formation of NC and for development of the autonomic nervous system (Britsch et al., 2001). *Sox10* is initially expressed by multi-potent progenitors that generate both neurons and glia in the periphery (Morrison et al., 1999; White et al., 2001; Kim et al., 2003; Corpening et al., 2011). Sox10+ progenitors have been documented in cranial ganglia, dorsal root ganglia, sympathetic ganglia, sciatic nerve, and enteric ganglia (Britsch et al., 2001; Paratore et al., 2001; Aquino et al., 2006; Walters et al., 2010; Corpening et al., 2011). As development of the peripheral nervous system proceeds, Sox10 becomes restricted to glial cells. We observed *Sox10* mRNA at high levels in regions of the LUT that coincided with known positions of NC-derived cell types (adrenal medulla) and autonomic peripheral ganglia (celiac ganglia, pelvic ganglia). In addition *Sox10* was also expressed in discrete cells within the bladder body and the urethra (Figure 3). The punctate distribution of individual Sox10+ cells in the urethra and bladder wall that we observed likely represents peripheral glial cells that accompany axonal processes in these structures since Sox10 is down-regulated in differentiating neurons. The images shown were allowed to develop for substantial periods of time in order to detect all possible Sox10+ cells present in the bladder and urethra. Samples developed for shorter time periods in BM purple substrate were also obtained (Figures 3D,E) and exhibited comparable distributions of cells in the bladder wall and urethra. Interestingly these under-developed samples revealed that the highest density of cells transcribing Sox10 is in a central region near the apex of the triangular pelvic ganglia. Previous analyses of NC-derived peripheral ganglia have not detected regional differences in intensity or distribution of Sox10 staining but generally have been more focused on neuronal markers that are expressed after Sox10 down regulates.

### Survey to identify genes expressed in fetal pelvic ganglia

Networks of TF expression that play critical roles in patterning and neurogenesis have been investigated in the central nervous system (Gray et al., 2004; Magdaleno et al., 2006; Fu et al., 2009). Some TFs that are essential for normal development of peripheral autonomic ganglia in the intestine including *Sox10*, *Phox2b*, and *Hand2* have been identified through analysis of disease models (Southard-Smith et al., 1998; Pattyn et al., 1999; D’Autreaux et al., 2007; Hendershot et al., 2007; Lei and Howard, 2011). However detailed knowledge of gene networks that participate in development of pelvic autonomic ganglia is lacking. And, to date, expression of only a handful of TFs [cJun (Nangle and Keast, 2009; Peddie and Keast, 2011), Stat (Bella et al., 2007), ERα, ERβ (Papka et al., 2001), cFOS (Fang et al., 2000), androgen receptor (Schirar et al., 1997)] has been identified in pelvic ganglia. To identify potential regulatory networks that are expressed during formation of pelvic neural elements, we screened a genome scale WISH library comprised of intact mouse 15.5dpc fetal UGTs available through the GUDMAP resource (McMahon et al., 2008; Mugford et al., 2009; Harding et al., 2011). Among 1379 gene expression patterns examined, we identified 155 genes that exhibited definitive expression within pelvic ganglia (Table 1). Among these we sought to identify that subset of genes encoding TFs. Two prior studies relied on the presence of DNA-binding domains as a criteria to identify TF genes (Gray et al., 2004; Lee et al., 2007). Both studies focused on the presence of DNA-binding domains as a criterion though each used a slightly different method to refine their selections. More recent efforts have focused this list to the most likely subset of DNA-binding transcriptional regulators based on consensus overlap of both lists (Yu et al., 2012). Using this selective list, we determined that 88 of the original 155 expressed in pelvic ganglia are TFs (asterisked gene names Table 1). Comprehensive details of gene name and *in situ* probe identifiers for samples in which pelvic ganglia expression was scored as definitely present or not detected by WISH in this survey are provided in Table S1 in Supplementary Material. Both male and female fetal UGTs were evaluated for expression of TFs. For those samples where pelvic ganglia expression was detected, technical replicates were evaluated for both genders however we did not detect any reproducible differences in distribution of WISH signal between genders. Images of all WISH samples with detectable pelvic ganglia expression have been posted to the GUDMAP consortium database for public access (www.gudmap.org).

**Table 1:** **Genes detected in pelvic ganglia at 15.5dpc by whole-mount *in situ* hybridization**.

Gene name	Probe no.	Gene name	Probe no.	Gene name	Probe no.
*4930555K19Rik*	MTF1122	*Kbtbd5*	MTF1668	*Rnf187*	MTF1390
*Ablim1*	MTF1346	*Kdm4a*	MTF1428	*Rnf41*	MTF1048
*Ahr	MTF1011	*Kdm4b	MTF1420	*Rnf44	MTF1049
*Aip*	MTF0733	*Kdm5b	MTF1089	*Rorc*	MTF0058
*Ankfy1*	MTF1529	*Kif1a*	MTF1178	*Rtn2	MTF0682
*Atf2	MTF0755	*Kif1b*	MTF1179	*Rtn4*	MTF0684
*Atf4	MTF0756	*Klf7	MTF0931	*Satb1*	MTF0706
*Atf5	MTF0785	*Klhl9*	MTF1553	*Sf1	MTF0869
*Bach2	MTF0582	*Lass5	MTF1983	*Sin3b*	MTF1846
*Bop1*	MTF0947	*Lcor*	MTF1923	*Six1	MTF0180
*Brca1*	MTF1518	*Limk1*	MTF1451	*Smad9	MTF2019
*Brd2*	MTF0221	*Lmo1	MTF0178	*Smarca4*	MTF0639
*Brd9*	MTF1959	*Lztr1*	MTF0783	*Smarcd1	MTF0643
*Cbl*	MTF1556	*Maff	MTF0555	*Snf8*	MTF1173
*Chd4*	MTF1557	*Mafg	MTF0556	*Sox10	MTF0511
*Cited2	MTF1133	*Mkrn2*	MTF0891	*Sox2	MTF0503
*Cnot7*	MTF1158	*Mllt4*	MTF1184	*Sox4	MTF0505
*Cops3*	MTF1309	*Mnt	MTF1193	*Sox9	MTF0510
*Cops6*	MTF1155	*Mpnd*	MTF1149	*Sqstm1	MTF0693
*Crip2*	MTF1826	*Mrps17*	MTF1490	*Srebf2*	MTF1206
*Cttn*	MTF0994	*Mxd1	MTF0799	*Stat3	MTF0773
*Cux2	MTF0744	*Myc	MTF1817	*Tacc2	MTF0635
*Dcun1d3*	MTF1816	*Myt1	MTF0902	*Tbx3*	MTF1311
*Dido1	MTF1296	*Myt1l	MTF0907	*Tcf4	MTF1939
*Dpf1	MTF1376	*Ncoa2	MTF0618	*Tcfap2b	MTF0778
*Dpf2	MTF1298	*Ndrg1*	MTF0964	*Tead1	MTF0596
*Dpf3*	MTF1503	*Ndrg2*	MTF0967	*Thbs3	MTF1516
*E4f1	MTF0923	*Ndrg3*	MTF0970	*Tomm6*	MTF0164
*Eea1*	MTF1538	*Ndrg4*	MTF0968	*Trim16*	MTF1065
*Eif4h*	MTF1933	*Nfe2l1	MTF0573	*Trim2*	MTF1098
*Ets2	MTF0400	*Nr2f1*	MTF0077	*Trim3*	MTF1056
*Fos	MTF0567	*Nr4a1	MTF0050	*Trim47*	MTF1426
*Foxk2	MTF1899	*Onecut1	MTF0151	*Trim68*	MTF1697
*Gata2	MTF0805	*Otp	MTF0191	*Trim8*	MTF1060
*Gata3	MTF0806	*Pax3	MTF0265	*Trim9*	MTF1396
*Gtf2a1	MTF1956	*Pcgf1*	MTF1700	*Usf1*	MTF0830
*Gtf3a	MTF1861	*Pcgf3	MTF1023	*Usf2	MTF0831
*Hand1	MTF0823	*Phf1	MTF1283	*Vps26b	MTF1493
*Hand2	MTF0972	*Phip*	MTF0241	*Zbtb16*	MTF1759
*Heyl	MTF0955	*Phox2b	MTF0363	*Zbtb22*	MTF1769
*Hivep2	MTF0689	*Plxna3*	MTF1335	*Zcchc14	MTF1545
*Hmbox1	MTF1211	*Plxnc1*	MTF1329	*Zfhx2*	MTF1525
*Hmgxb3	MTF2008	*Pogz*	MTF1874	*Zfhx3	MTF0141
*Hmx1	MTF0006	*Pou2f2	MTF0219	*Zfp318	MTF1770
*Hoxc10	MTF0105	*Pou3f3	MTF0210	*Zfp326	MTF1771
*Hoxc8	MTF0319	*Pou6f1	MTF0215	*Zfp426*	MTF1369
*Hoxd10	MTF0106	*Prox1	MTF0329	*Zfp467	MTF0898
*Hoxd3*	MTF0135	*Prrx1	MTF0196	*Zfp612	MTF1382
*Ing4*	MTF1285	*Rnf112	MTF0870	*Zmynd8	MTF1571
*Irf5	MTF0560	*Rnf113a1	MTF1052	*Znrf1	MTF1051
*Isl1	MTF1380	*Rnf130*	MTF0900	*Zscan21*	MTF0908
*Jund	MTF0551	*Rnf14*	MTF1033		

**Figure 4:**
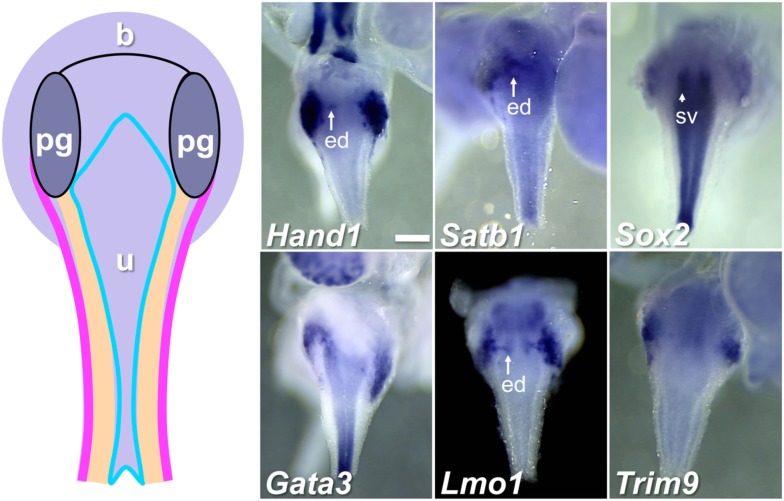
**Genes expressed in pelvic ganglia and urethral epithelium in the fetal mouse LUT**. A schematic diagram of LUT represents dorsal views of the urethra (u), including the epithelial layer (blue), mesenchymal layer (peach), muscular layer (pink), and pelvic ganglia (pg). Distinct expression patterns in the urethral epithelium and pelvic ganglia are evident in dorsal views at 15.5dpc for a subset of WISH samples surveyed. All images are 50× magnification with a 300 μm scale bar in the first panel. ed, Ejaculatory duct (arrow); sv, seminal vescicles (arrowhead). Gene abbreviation/MGI gene ID/gender of sample: *Hand1*/103577/male; *Satb1*/105084/male; *Sox2*/98364/female; *Gata3*/95663/male; *Lmo1*/102812/male; *Trim9*/2137354/female.

To begin to categorize gene expression patterns among the total 155 identified within the pelvic ganglia, we noted those genes that were expressed not only in pelvic ganglia but also in LUT epithelium (Table 2). Representative images of expression patterns observed for genes exhibiting both pelvic ganglia and LUT epithelial expression including *Hand1*, *Satb1*, *Sox2*, *Gata3*, *Lmo1*, and *Trim9* are shown in Figure 4. *Hand1*, *Satb1*, *Lmo1*, and *Trim9* were expressed at high levels in pelvic ganglia and discrete domains within medial urethral structures of the ejaculatory duct in males or lower vagina in females. *Sox2* and *Gata3* exhibited much lower expression in pelvic ganglia with significantly stronger expression in urethral epithelium.

**Table 2:** **Genes detected in pelvic ganglia and LUT epithelium**.

Gene name	Probe no.	Gene name	Probe no.	Gene name	Probe no.
*4930555k19Rik*	MTF1122	*Hoxc8*	MTF0319	*Rnf113a1*	MTF1052
*Ablim1*	MTF1346	*HoxD3*	MTF0135	*Rnf130*	MTF0900
*Ahr*	MTF1011	*Irf5*	MTF0560	*Satb1*	MTF0706
*Atf5*	MTF0785	*Lmo1*	MTF0178	*Sox2*	MTF0503
*Baz2a*	MTF0233	*Mafg*	MTF0556	*Sqstm1*	MTF0693
*Dcun1d3*	MTF1816	*Lcor*	MTF1923	*Thbs3*	MTF1516
*Bop1*	MTF0947	*Myt1l*	MTF0371	*Trim16*	MTF1065
*Cnot7*	MTF1158	*Nfe2L1*	MTF0573	*Trim2*	MTF1098
*Dpf3*	MTF1503	*Pax3*	MTF0265	*Trim9*	MTF1396
*Gata3*	MTF0806	*Phip*	MTF0241	*Zbtb16*	MTF1759
*Gtf3a*	MTF1861	*Pou2f2*	MTF0219	*Zfp426*	MTF1369
*Hand1*	MTF0823	*Rnf187*	MTF1390	*Zfp6339*	MTF1886

**Figure 5:**
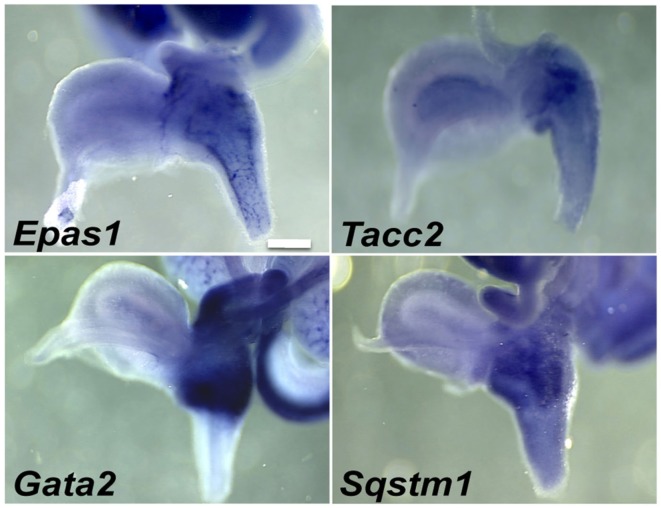
**Pelvic ganglia and urethral mesenchyme expression of genes in the fetal LUT identified by WISH**. Lateral views of gene expression patterns detected for a subset of genes expressed in both pelvic ganglia and urethral mesenchyme. All images are 50× magnification with a 300 μm scale bar. Gene abbreviation/MGI gene ID/gender of sample: *Epas1*/109169/female; *Tacc2*/1928899/male; *Gata2*/95662/female; *Sqstm1*/107931/female.

A second category of potential regulatory genes expressed in pelvic ganglia also exhibited expression in LUT mesenchyme (Table 3). Some of these genes exhibited strong pelvic ganglia expression accompanied by more modest expression in discrete urethral domains (Figure 5). Specifically *Tacc2* and *Sqstm1* exhibited diffuse regional expression in the ventral urethra at 15.5dpc. Other genes, like the TF *Gata2*, exhibited strong regional expression throughout the upper third of the urethra including the pelvic ganglia. A few genes, such as *Epas1*, exhibited distinctive expression throughout the entire urethra, bladder neck, and pelvic ganglia. Whether these regional expression patterns are involved in dorsal-ventral patterning, development of vasculature or muscle remains to be determined through co-localization with cell-type specific markers and evaluation of mutant alleles that are being constructed (Harding et al., 2011; Skarnes et al., 2011).

**Table 3:** **Genes detected in pelvic ganglia and LUT mesenchyme**.

Gene name	Probe no.	Gene name	Probe no.	Gene name	Probe no.
*Aip*	MTF0733	*Heyl*	MTF0955	*Rtn4*	MTF0684
*Bach2*	MTF0582	*Hivep2*	MTF0689	*Smarcd1*	MTF0643
*Crip2*	MTF1826	*Klf7*	MTF0931	*Sqstm1*	MTF0693
*Cux2*	MTF0744	*Kdm4a*	MTF1428	*Tacc2*	MTF0635
*Epas1*	MTF0540	*Meis1*	MTF0118	*Tead1*	MTF0596
*Fos*	MTF0567	*Myc*	MTF1817	*Usf1*	MTF0830
*Gata2*	MTF0805	*Ncoa2*	MTF0618	*Usf2*	MTF0831
*Gtf2a1*	MTF1956	*Ndrg2*	MTF0967	*Vps26b*	MTF1493
*Hand2*	MTF0972	*Pcgf3*	MTF1023	*Zmym3*	MTF0879

**Figure 6:**
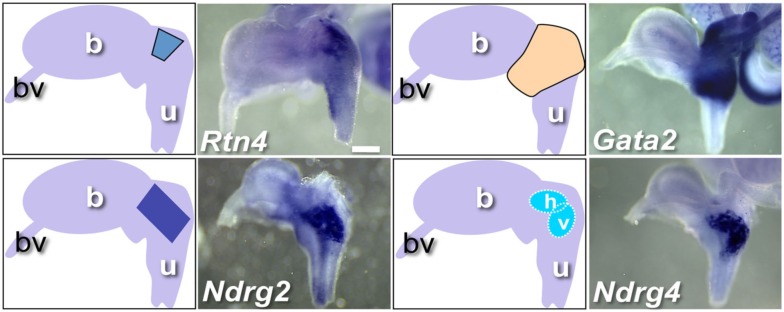
**WISH Gene expression patterns within and around fetal pelvic ganglia**. Representative lateral views of individual gene expression patterns that exhibit distinct regional expression patterns within the pelvic ganglia are shown. Accompanying diagrams emphasize area of highest LUT expression detected for each gene. All images are 50× magnification with a 300 μm scale bar. Gene abbreviation/MGI gene ID/gender of sample: *Rtn4*/1915835/male; *Gata2*/95662/female; *Ndrg2*/1352498/female; *Ndrg4*/2384590/female. (b, bladder; bv, blood vessel; u, urethra; h, horizontal domain; v, vertical domain).

During imaging and analysis of fetal WISH samples, we observed that several genes exhibited expression confined to distinct regional domains within and around the pelvic ganglia. *Rtn4*, *Ndrg2*, *Gata2*, and *Ndrg4* illustrate such restricted patterns (Figure 6). *Gata2* probes detected expression at very high levels in a banded pattern throughout the bladder neck and upper urethra including the pelvic ganglia. This contrasted to very restricted expression observed for *Rtn4* and *Ndrg2* within the pelvic ganglia. *Ndrg4* exemplifies a subset of genes that showed very highly localized expression within clusters of cells internal to the pelvic ganglia in two oval domains that were either parallel to the axis of the bladder neck (horizontal) or vertically aligned to the urethral epithelium (vertical). Several other genes including *Rnf31*, *Kif1b*, and *Mitc1* exhibited expression in these oval domains albeit at much lower levels than *Ndrg4* (data not shown, WISH images posted to www.gupmap.org). Given that TFs play a prominent role in patterning the developing central nervous system, the regulatory factors identified here may play essential roles in specifying neuronal subtypes or may be required for the maintenance of different cell populations within the pelvic ganglia as this structure matures.

**Figure 7:**
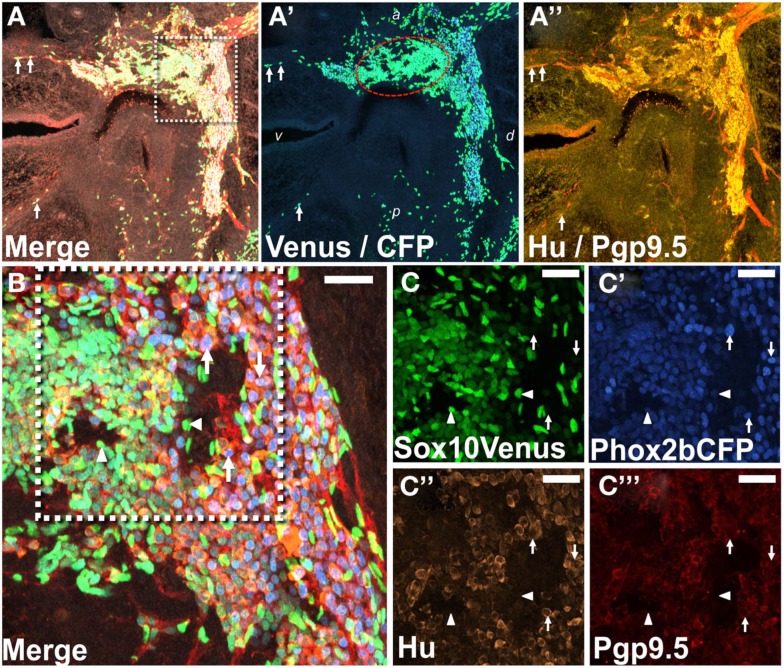
**Regional segregation of NC-derived progenitors and neurons within fetal pelvic ganglia**. **(A)** Cryosection through the lateral sagittal plane of pelvic ganglia collected from *Phox2b*-H2BCFP, *Sox10*-H2BVenus double transgenic female fetal mouse stained for PGP9.5 and Hu shows the overall triangular shape of the pelvic ganglia at 15.5dpc. Merged confocal image at 200× magnification captures nuclear Sox10-H2BVenus (green), nuclear Phox2b-H2BCFP (blue), cytoplasmic PGP9.5 (red), and Hu (gold). Differentiating neurons in the bladder wall identified by residual nuclear H2BVenus expression are co-labeled by up-regulation of cytoplasmic PGP9.5 (arrows). **(A′)** Confocal image showing channels only for Venus and CFP from the cryosection presented in **(A)** is shown. An oval domain comprised of densely packed Sox10+ cells (green nuclei, encircled by red dotted line) is evident with Phox2b+ neurons (blue nuclei) being most numerous outside this region. **(A″)** A confocal image shows channels for PGP9.5 and Hu from the cryosection presented in **(A)**. PGP9.5 (red) labels numerous cell bodies at the dorsal aspect of the pelvic ganglia, a small area of differentiating neurons nearest the anterior bladder neck, and extrinsic nerve fibers entering the ganglia. Hu (gold) labels numerous cell bodies with the greatest density being around the perimeter of the pelvic ganglia. **(B)** Higher magnification confocal image (400×) from the boxed region in **(A)** shows high density of H2BVenus+ progenitors in the core of the oval progenitor domain with Phox2b+, PGP9.5+, Hu+ neurons clustered at the dorsal aspect of the pelvic ganglia. Single confocal channels at 630× magnification from boxed area in **(B)** are shown for *Sox10*-H2BVenus [**(C)**, green], *Phox2b*-H2BCFP [**(C′)**, blue], Hu+ neuronal soma [**(C″)**, gold], PGP9.5+ differentiating neurons [**(C‴)**, red]. Progenitor cells labeled by H2BVenus nuclear fluorescence (arrowheads) exhibit low or no expression of *Phox2b*-H2BCFP and no expression of PGP9.5 and Hu. Differentiating neurons lack H2BVenus label, exhibit bright *Phox2b*-H2BCFP nuclear fluorescence as well as cytoplasmic Hu and PGP9.5 labeling (arrows). Dorsal (d), ventral (v), anterior (a), and posterior (p) orientations are indicated on **(A′)**. Scale bar: 25 μm.

The tight clustering of cells labeled by *Ndrg4* within a central domain in the developing pelvic ganglia is reminiscent of processes that occur in development of ciliary ganglia, sympathetic ganglia, and dorsal root ganglia. In these ganglia differentiating neurons and progenitors segregate into regional domains that are recognizable on the basis of markers that label differentiated neurons versus those that label progenitors (Muller and Rohrer, 2002; Callahan et al., 2008). In order to determine whether a similar segregation process might account for the localized expression of *Ndrg4* and other TFs in the oval domains we observed in developing pelvic ganglia, we evaluated the distribution of Sox10+ and Phox2b+ cells in LUT tissues of *Sox10-*H2BVenus, *Phox2b-*H2BCFP double transgenic fetal mice. The *Sox10*-H2BVenus BAC transgenic line recapitulates expression patterns of the endogenous protein and thus labels NC-derived progenitors during development as well as peripheral glia in adult tissues (Corpening et al., 2011). Conversely the *Phox2b-*H2BCFP BAC transgenic is up-regulated and maintained at high levels in peripheral neurons (Corpening et al., 2008). Concurrent IHC staining for PGP9.5 and Hu was applied to these samples to confirm the location of differentiating neurons since the distribution of Phox2b has previously not been examined at high resolution in the developing pelvic ganglia. Confocal images of sagittal sections through the LUT revealed the pelvic ganglia as a strongly PGP9.5/Hu+ triangular structure consistent with prior reports of the overall triangular shape of this ganglia in adult mice (Figure 7; Wanigasekara et al., 2003). While the majority of PGP9.5+ cells resided within the structure of the pelvic ganglia, a few PGP9.5+ cells were evident within the dorsal and ventral aspects of the bladder wall (Figure 7A). Interestingly, within the pelvic ganglia at 15.5dpc we observed noticeable oval domains strongly positive for *Sox10*-H2BVenus expression (Figure 7A′). Such domains were weakly *Phox2b*-H2BCFP+, which would be consistent with progenitor cells, while nearby cells were strongly Phox2b-H2BCFP+, PGP9.5+, and Hu+ consistent with neuronal identity (Figure 7B). The regional density of Sox10+ cells within oval domains was observed in multiple samples from *Sox10-*H2BVenus, *Phox2b-*H2BCFP double transgenic embryos and appears to coincide with the regions labeled by WISH for *Ndrg4* and other genes. The clustering of large numbers of strongly Sox10+ cells in the interior of the pelvic ganglia demonstrates that NC-derived progenitors are present in this region at 15.5dpc and further supports the notion that regional expression patterns observed in WISH are indicative of domains with the developing pelvic ganglia at this stage.

**Figure 8:**
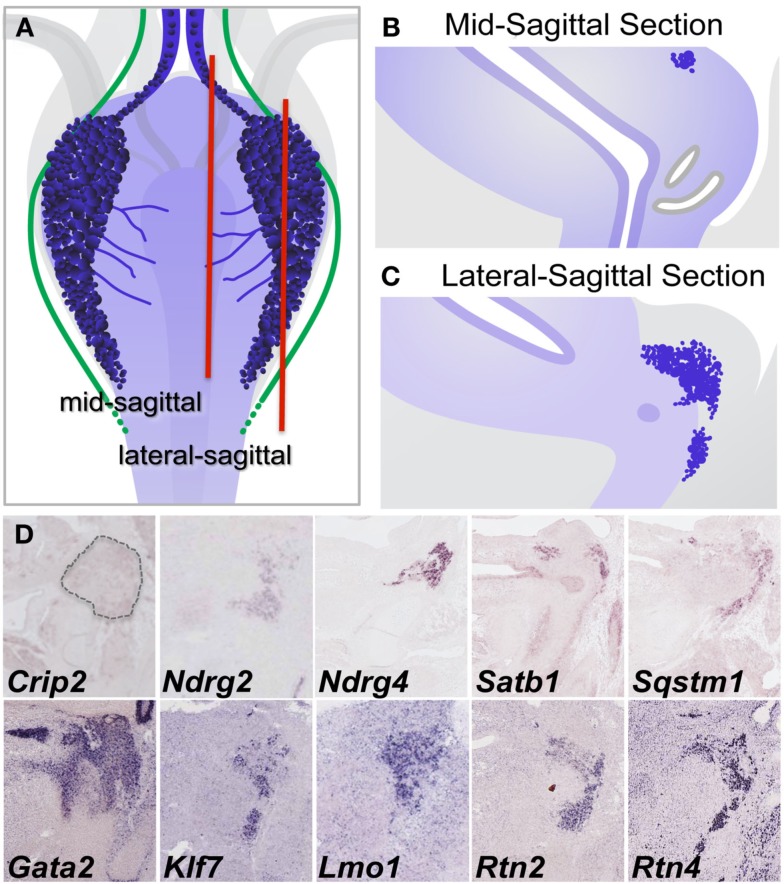
**Validation of gene expression patterns in pelvic ganglia by sectional *in situ* hybridization (SISH). (A)** Schematic diagram of 15.5dpc fetal mouse LUT viewed dorsally illustrates pelvic ganglia (dark blue clusters), neurites extending from pelvic ganglia across the dorsal urethral surface (blue lines), and axonal processes (green lines) relative to LUT structures. Red vertical lines indicate the relative planes of cryosections taken through mid-sagittal **(B)** and lateral sagittal regions **(C)** for SISH processing. Colorometric micrographs of SISH signals obtained in cryosections for individual genes **(D)**. Images for *Crip2*, *Ndrg4*, *Satb1*, and *Sqstm1* are from data deposited at the GUDMAP database (probe IDs: MGI:3507380, MGI:3506573, MGI:3506384, MGI:3506375, respectively). Images for Ndrg2, *Gata2*, *Klf7*, *Lmo1*, *Rtn2*, and *Rtn4* derive from data posted on Eurexpress.org (probe IDs: euxassay_010581, section 14; euxassay_018037, section 14; euxassay_003485, section 16; euxassay_017874, section 07; euxassay_018264, section 14; and euxassay_004344, section 14, respectively). Dotted line outlines the pelvic ganglia border in SISH panel for Crip2 as the expression for this gene is not homogeneous within cells but was observed as faint punctate signal within some cells and not in others.

To confirm expression within pelvic ganglia and attempt to better define the distribution of individual gene transcripts within pelvic ganglia, sectional *in situ* hybridization (SISH) was performed (Figure 8). In addition, we evaluated public SISH data posted to Eurexpress for the distribution of these transcripts in the LUT (Diez-Roux et al., 2011). In sagittal tissue sections captured close to the mid-line, colorimetric ISH signal was present for *Crip2*, *Ndrg2*, *Ndrg4*, *Satb1*, and *Sqstm1* in proximity to the nephric duct and ureter insertion sites where the bladder neck joins the urethra (Figure 8D). *Gata2* expression was observed throughout the entire area of the bladder neck and was not restricted to pelvic ganglia. Expression of *Ndrg2*, *Ndrg4*, *Klf7*, *Lmo1*, *Rtn2*, and *Rtn4* was present not only at high levels in the pelvic ganglia (Figure 8D) but also in other aspects of the developing nervous system (data not shown, public images accessible at www.eurexpress.org). *Crip2* signal was observed in a diffuse punctate distribution within the pelvic ganglia but was also present in other tissues nearby. The expression patterns detected by SISH are consistent with expression of these genes within pelvic ganglia.

### Analysis of gene expression in post-natal pelvic ganglia

**Figure 9:**
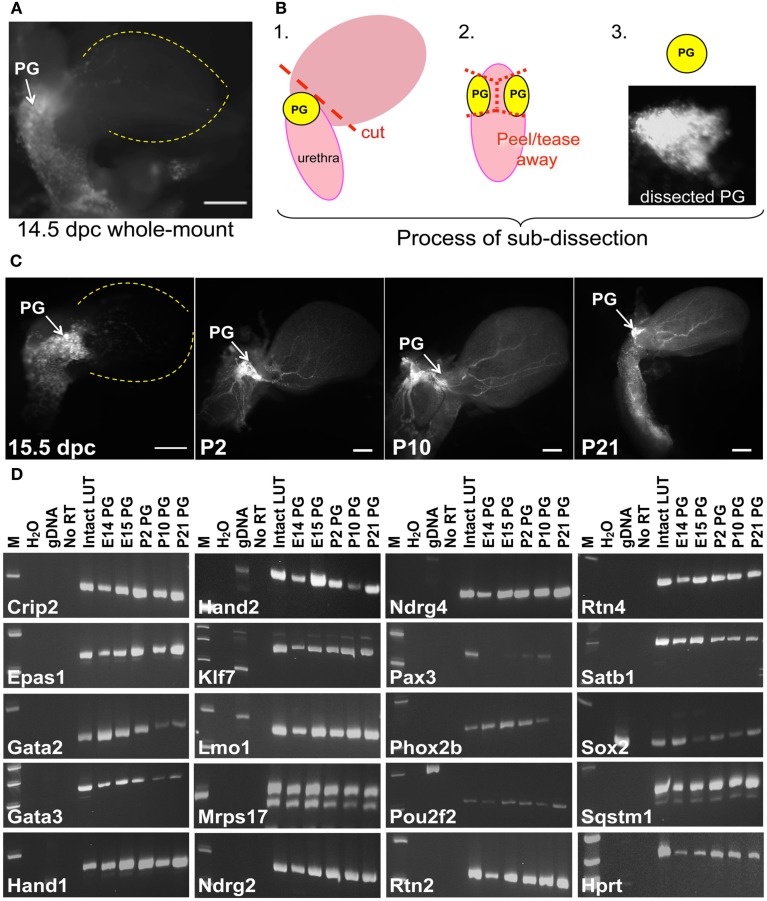
**Identification of genes that maintain expression in pelvic ganglia across multiple developmental stages**. **(A)** NC derivatives labeled by *Sox10* expression are evident in pelvic ganglia, bladder wall, and urethra of a *Sox10*-H2BVenus transgenic embryo viewed laterally at 14.5dpc. **(B)** Schematic diagram illustrating the dissection technique to harvest pelvic ganglia based on *Sox10*-H2BVenus transgene fluorescence. **(C)** Relative size, shape, and position of pelvic ganglia compared to the overall bladder morphology are illuminated by *Sox10*-H2BVenus expression in lateral images of 15.5dpc, P2, P10, P21 lower urinary tracts. Scale bar = 1000 μm. **(D)** Gel electrophoresis images of RT-PCR products relative to a standard molecular weight marker identifies individual genes expressed in fetal development and post-natal maturation of pelvic ganglia. Sample order: no template water control, genomic DNA control, no RT control (15.5dpc RNA sample), 15.5dpc intact LUT (consisting of distal ureter, bladder, urethra, pelvic ganglia, and genital tubercle) and *Sox10*-H2BVenus pelvic ganglia RNA isolates harvested at 14.5dpc, 15.5dpc, P2, P10, and P21. A housekeeping control gene (*Hprt*) is included for comparison.

Multiple TFs are essential for patterning distinct regions of the central nervous system (Sham et al., 1993; Barrow et al., 2000; Tomotsune et al., 2000; Kitaguchi et al., 2001) and for initiating and maintaining neurogenesis in the adult CNS (Gao et al., 2011; Jacquet et al., 2011; Karalay et al., 2011; Li et al., 2011). The gene expression patterns we observed in fetal LUT at 15.5dpc could represent transient transcription needed during development but might also be necessary for maintenance of neuronal and glial lineages as the LUT matures. To evaluate this possibility, we examined temporal expression for a subset of genes identified in the initial WISH screen. Pelvic ganglia were visualized by whole-mount fluorescence imaging of *Sox10*-H2BVenus transgene expression. Fluorescence expressed from the *Sox10*-H2BVenus transgene facilitates collection of pelvic ganglia and avoids surrounding tissues. Pelvic ganglia were imaged and dissected at five discrete stages [14.5 and 15.5dpc, Post-natal day 2 (P2), P10, and P21] and total RNA was isolated for analysis by RT-PCR. At all stages the pelvic ganglia were strongly labeled by *Sox10*-H2BVenus expression, which provided a perspective of the size and position of the pelvic ganglia relative to the bladder at multiple stages (Figures 9A–C). At 14.5dpc the pelvic ganglia are elongated clusters that flank the bladder neck and extend more than half way down either side of the pelvic urethra. During post-natal maturation these ganglia condense into the better-known triangular shape that is smaller compared to the overall size of the mature bladder.

To determine whether genes detected in the initial WISH screen maintained their expression at multiple points in pelvic ganglia ontogeny, RT-PCR was implemented using both RNA from intact LUT and from dissected pelvic ganglia. To ensure specificity of the RT-PCR assays oligonucleotide primers were designed to extend from 3′ untranslated regions into adjacent exons so that amplification from genomic DNA was avoided. In addition, primers were positioned to avoid homologous regions between related gene family members (Table S2 in Supplementary Material). The assays used only produced PCR products from cDNA with the exception of *Sox2*, which lacks introns (Figure 9). These gene-specific primers were used for RT and PCR detection of individual transcripts in pelvic ganglia RNA isolates at each of five stages. Control samples run in parallel consisted of 15.5dpc intact LUT RNA that included distal ureter, bladder, pelvic ganglia, urethra, and genital tubercle as well as genomic DNA (Figure 9D). All RT-PCR samples for an individual gene were reverse transcribed and PCR amplified in parallel then evaluated by gel electrophoresis to confirm appropriate product size. All of the genes initially detected by *in situ* hybridization were also detected by RT-PCR at 15.5dpc in multiple biological and technical replicates. While the majority of genes examined produced robust RT-PCR product, *Pax3* was not detected at 14.5dpc and product for this gene was barely discernable at 15.5dpc consistent with the extremely low WISH signal. *Pax3* RT-PCR product was still present at P10 but was no longer detectable at P21. The other genes evaluated were detected at all the stages examined, although the intensity of bands obtained for *Gata2*, *Gata3*, and *Phox2b* suggests that these factors diminish by P21. Intensity of RT-PCR product also varied noticeably for *Hand2*. While this could be due to variations in input cDNA between samples, previous temporal studies of *Hand2* expression in the enteric nervous system have shown that levels of this factor peak at 14.5dpc during fetal neurogenesis (D’Autreaux et al., 2007). The confirmed expression of these regulatory genes and their maintenance over a significant time frame in fetal development and post-natal maturation of pelvic ganglia leads to obvious questions about their functions in this structure.

## Discussion

It is widely accepted that sacral autonomic ganglia in the LUT derive from progenitors that originate at the NC and migrate into the region of the cloaca during organogenesis to form the pelvic ganglia. However the processes that control development of sacral autonomic ganglia and innervation of the LUT have not been described to date. Identification of genes that participate in this process is one means to gain an entry point into developmental pathways that will not only provide baseline information for future interrogation of gene function but may lead to discovery of relevant disease genes. In this report we have identified 155 murine genes that exhibit expression in pelvic ganglia including 88 TFs of which 82 have not been previously been linked to pelvic ganglia. Regional differences in expression patterns of these factors within the pelvic ganglia suggest progenitors and differentiating neurons are regionally segregated in early stages of development. Temporal analysis of expression for a subset of these genes demonstrates that many maintain their expression from early fetal organogenesis of the LUT through to post-natal maturation of pelvic ganglia. Given the clear role played by TFs in induction and patterning of the central nervous system as well as recent studies that demonstrate TFs play an essential part in determining cell fate decisions (Collombat et al., 2003; Hang and Stein, 2011; Hu He et al., 2011; Liu et al., 2011; Pan and Wright, 2011), the genes identified here should be prioritized for future studies of sacral innervation.

As a platform for annotating gene expression in the LUT, we provide an initial spatial distribution of neural elements in the fetal mouse LUT based on IHC and histochemical staining at 15.5dpc. Our identification of large numbers of PGP9.5+ cells visible as a dense network of neural fibers present in the developing bladder wall suggests that neural connections are being made during expansion and maturation of the bladder wall. Moreover the marked difference between cell numbers labeled by PGP9.5 immunoreactivity and axonal projections labeled by VAChT histochemistry suggests that a large number of PGP9.5+ cells are not cholinergic. This result raises several interesting questions: What cell types derive from the PGP9.5+ cells present in the medial wall and bladder dome at 15.5dpc? Is the broad PGP9.5+ expression representative of other previously unrecognized neuronal lineages at this stage of development given the documented role for PGP9.5 in neuronal development and synaptic connectivity (Sakurai et al., 2006; Cartier et al., 2009)? What are the relationships between VAChT+ axonal processes and the PGP9.5+ cells in the bladder wall if any? We also observed PGP9.5+ neuronal cell bodies in the bladder wall by sectional analysis at 15.5dpc (Figure 7). In the adult mouse neuronal soma have not previously been reported in the detrusor (Yan and Keast, 2008), but intramural ganglia are present in rats at P0 that then decline in numbers postnatally (Iuchi et al., 1994; Alian and Gabella, 1996; Zvarova and Vizzard, 2005). Whether derivatives of the early PGP9.5+ cells we have observed survive in the adult bladder and what connections they have with other cell types remains to be determined. These questions await future analyses of discrete lineages in a comprehensive timeline of the developing bladder wall.

*Sox10* expression marks multi-potent NC-derived progenitors in early stages of development and is maintained in glia as peripheral ganglia mature (Bremer et al., 2011). In this study we demonstrate that *Sox10* expression is present in a large number of cells in the LUT at 15.5dpc. The expression pattern observed was detected with an *in situ* probe that contained homology to other SoxE box proteins including *Sox8* and *Sox9*. However, it is unlikely that any of the Sox10 WISH signal is due to cross-hybridization to other Sox gene family members because riboprobes are capable of discriminating between gene family members when RNase treatment is performed after hybridization to degrade any mismatched hybrid strands as was done in this case. Neither *Sox8* nor *Sox9* probes exhibited intense staining of pelvic ganglia or any signal throughout the urethra comparable to what was observed for the *Sox10* WISH probe. In addition, the expression pattern we document for the *Sox10*-H2BVenus transgene in LUT (Figure 8) is comparable to the *Sox10* WISH probe signal and thus supports the specificity of the WISH signal detected. Surprisingly few studies have previously visualized NC-derived progenitors in the developing murine LUT (Stewart et al., 2008; Abler et al., 2011; Mundell et al., 2012). Given the large numbers of NC-derived Sox10+ cells in the LUT, studies to investigate the lineages that derive from these progenitors and their inductive effects in this organ system should be of interest to the urologic community, particularly in light of bladder dysfunction in neurocristopathies like Spina bifida.

Prior to the presented analysis, few studies describing expression of TFs in pelvic ganglia had been published. Our survey identified a large number of potential TFs expressed in pelvic ganglia at 15.5dpc. If a strict requirement for DNA-binding is imposed on this subset the list narrows to 88 TFs. Similar surveys to investigate glial-specific TFs in the developing central nervous system have also identified large numbers of genes in developing glia. Given the potential complexity of neuronal and glial cell types in adult pelvic ganglia identification of a large number of potential regulatory TFs at a single stage in development is not surprising. This entry-level screen provides a significantly expanded candidate list of genes that may now be pursued in functional studies to determine if these genes are required for maintaining neural progenitors, for promoting terminal differentiation of neurons, or for balancing segregation of lineages.

One conclusion from our study is that gene expression within the developing pelvic ganglia is not homogeneous. We documented expression patterns that appear to label a subset of cells within the pelvic ganglia at 15.5dpc. Some of these subpopulations are labeled by TF expression (*Hand1*, *Gata2*, and *Lmo1*) but others (*Rtn4*, *Ndrg2*, and *Ndrg4*) have not proven to be TFs and were simply included in the initial survey based on the presence of predicted DNA-binding domain in their protein sequence (Gray et al., 2004). Despite not exhibiting DNA-binding activity, *Rtn4*, *Ndrg2*, and *Ndrg4* are of particular interest because of their highly localized expression in pelvic ganglia and also because of their known roles in other aspects of neural development. The *Rtn4* locus encodes Nogo, a major myelin-derived axon growth inhibitor that plays a role in axonal fasciculation and is also expressed on growing axons during development (Hunt et al., 2002). *Ndrg2* encodes a member of the N-myc downstream-regulated gene (NDRG) family, is expressed in differentiating astroglial cells in the CNS and appears to play a role in astroglial activation (Shen et al., 2008; Takeichi et al., 2011). Perhaps the most remarkable expression pattern we describe is that of *Ndrg4* that labels core clusters of cells within the developing pelvic ganglia. LUT phenotypes for *Ndrg4* knockout mice have not been reported but these mutants do exhibit cognitive deficits and they are less tolerant of ischemic stress after cerebral infarction (Yamamoto et al., 2011). Whether TF or not, the expression patterns of these genes suggests regional domains within pelvic ganglia that have not previously been described.

Interestingly the distribution of Sox10 and Phox2b relative to neuronal markers PGP9.5 and Hu also supports the tenant that the pelvic ganglia contains subdomains of progenitor cells and developing neurons. Both WISH and transgene expression indicate that *Sox10* expression labels the largest total area of pelvic ganglia. However sectional analysis revealed that the highest density of Sox10+ progenitors, identified by H2BVenus nuclear transgene label, resides in an oval domain perpendicular to the urethra. Differentiating neurons labeled by Phox2b, PGP9.5, and Hu are clustered in greatest numbers outside this domain of high-density Sox10+ progenitors. Similar segregation of progenitors and differentiating neurons has previously been observed in sympathetic ganglia, ciliary ganglia, and dorsal root ganglia, all of which derive from NC-progenitors (Muller and Rohrer, 2002; Callahan et al., 2008; Tsarovina et al., 2008; Nishi et al., 2010). Identification of a progenitor domain within the developing pelvic ganglia is not entirely unexpected particularly given gene expression patterns in ciliary ganglia where neurons initially appear on the perimeter of an undifferentiated core in the developing ganglia (Muller and Rohrer, 2002). As development proceeds expression of neuronal markers becomes more uniform across the ciliary ganglia (Muller and Rohrer, 2002). Similar restructuring of the pelvic ganglia through development may occur as well. An expression profile of the TFs identified in this study relative to the regional placement of progenitors and neurons within the pelvic ganglia may identify factors that control this process. Initial studies that visualized calcium transients during development of sympathetic ganglia suggest that calcium signaling is somehow involved in the regional segregation of progenitors and neurons (McKinney and Kulesa, 2011).

While our survey has identified transcription of numerous potential regulatory factors based on *in situ* and RT-PCR, it is possible that the presence of mRNA does not necessarily imply protein expression due to post-transcriptional regulatory mechanisms that may be in place. Transcription of other TFs, like *Tcf4* and *Olig1* that function in gliogenesis, is known to occur several days in advance of protein expression (Lu et al., 2000; Fu et al., 2009). Definitive correlation between transcription and protein expression will require close attention to the specificity of any antibodies that are deployed for such studies as cross-reactivity between DNA-binding domains of TFs can complicate specific detection of closely related gene family members.

In summary, we have screened an extensive expression resource to identify potential regulatory genes that are expressed in the pelvic ganglia. This is the first comprehensive survey to identify genes that participate in development of LUT innervation. Our efforts have identified 82 genes that have not previously been identified in fetal mouse pelvic ganglia and demonstrate that some of these are maintained in the post-natal ganglia. Several of these genes exhibited expression in recognizable domains within the pelvic ganglia suggesting that there may be regional segregation of cell types within this structure. Future studies to identify those genes expressed in progenitors relative to differentiating neurons will be of interest for investigators seeking to better understand development of sacral innervation. Moreover, overlapping expression of individual TFs may impart neuronal identity analogous to domain specific expression conferred by TFs expressed in the spinal cord. Identifying the factors, whether TF or not, that control development and maintenance of pelvic ganglia is relevant for understanding bladder control and may benefit efforts to innervate artificial bladder scaffolds or repair damaged pelvic innervation.

## Conflict of Interest Statement

The authors declare that the research was conducted in the absence of any commercial or financial relationships that could be construed as a potential conflict of interest.

## Supplementary Material

The Supplementary Material for this article can be found online at: http://www.frontiersin.org/Autonomic_Neuroscience/10.3389/fnins.2012.00130/abstract

Supplementary Table S1 **Compilation of WISH samples scored for gene expression in pelvic ganglia**. A summary list of WISH samples surveyed that exhibited either definitive expression, potential expression (very weak), or clearly not detected in pelvic ganglia of mouse fetal LUT is provided. The gene symbol, MTF number, gene description, MGI gene ID, Ensembl gene ID, and MGI probe ID is listed for each sample viewed and scored. Transcription factor genes are indicated in column G.

Supplementary Table S2 **RT-PCR primers utilized for each gene in the RT-PCR analysis**.
